# Estimating Sugarcane Yield Potential Using an In-Season Determination of Normalized Difference Vegetative Index

**DOI:** 10.3390/s120607529

**Published:** 2012-06-04

**Authors:** Josh Lofton, Brenda S. Tubana, Yumiko Kanke, Jasper Teboh, Howard Viator, Marilyn Dalen

**Affiliations:** 1 Macon Ridge Research Station, Louisiana State University AgCenter, Winnsboro, LA 71295, USA; E-Mail: jlofton@agcenter.lsu.edu; 2 School of Plant, Environmental, and Soil Science, Louisiana State University AgCenter, Baton Rouge, LA 70801, USA; E-Mails: ykanke1@tigers.lsu.edu (Y.K.); mdalen1@tigers.lsu.edu (M.D.); 3 Carrington Research Extension Center, North Dakota State University, Carrington, ND 58421, USA; E-Mail: JTeboh@agcenter.lsu.edu; 4 Iberia Research Station, Louisiana State University AgCenter, Jeanerette, LA 70544, USA; E-Mail: HViator@agcenter.lsu.edu

**Keywords:** sugarcane, nitrogen, NDVI, precision agriculture

## Abstract

Estimating crop yield using remote sensing techniques has proven to be successful. However, sugarcane possesses unique characteristics; such as, a multi-year cropping cycle and plant height-limiting for midseason fertilizer application timing. Our study objective was to determine if sugarcane yield potential could be estimated using an in-season estimation of normalized difference vegetative index (NDVI). Sensor readings were taken using the GreenSeeker® handheld sensor from 2008 to 2011 in St. Gabriel and Jeanerette, LA, USA. In-season estimates of yield (INSEY) values were calculated by dividing NDVI by thermal variables. Optimum timing for estimating sugarcane yield was between 601–750 GDD. In-season estimated yield values improved the yield potential (YP) model compared to using NDVI. Generally, INSEY value showed a positive exponential relationship with yield (*r^2^* values 0.48 and 0.42 for cane tonnage and sugar yield, respectively). When models were separated based on canopy structure there was an increase the strength of the relationship for the erectophile varieties (*r^2^* 0.53 and 0.47 for cane tonnage and sugar yield, respectively); however, the model for planophile varieties weakened slightly. Results of this study indicate using an INSEY value for predicting sugarcane yield shows potential of being a valuable management tool for sugarcane producers in Louisiana.

## 1. Introduction

Sugarcane (*Saccharum officinarum*) is an integral part of Louisiana's economy and culture, with an annual economic value exceeding $US 2 billion [1]. In recent decades significant yield increases have been attributed to the addition of N fertilizer beyond any other agricultural input [2]. Nitrogen (N) is one of the most important crop growth factors, influencing both productivity and crop quality. Therefore, utilizing methods that can more accurately determine N rate recommendations is essential to maintain agronomic productivity [3].

Sugarcane is a semi-perennial crop, which can be harvested annually up to five years without replanting; the first harvested crop is termed plant cane and stubble cane for each successive harvest. In Louisiana, the sugarcane growth season is shorter, typically only nine months, compared to other sugarcane growing regions, where it can be >12 months. The sugarcane growth season consists of planting in August or September, the sugarcane crop is terminated during the winter months due to freezing temperatures, growth is recommenced in late February to early March, and harvested the following October through December. During this growth season the sugarcane crop undergoes four distinct growth stages consisting of emergence, tillering, grand growth, and maturation, each of which typically lasts 1 to 3 months. In Louisiana, a planting cycle typically last three to four growth seasons prior to replanting. These long growth cycles combined with the shorter growth makes accurate N rate recommendations that optimize yields and minimize environmental impacts difficult. Worldwide N recommendations for sugarcane production are dependent on climate, crop age, length of growth cycle, plant characteristics, and soil characteristics [3]. However, currently for Louisiana sugarcane production N rate recommendations are dependent on crop age, either plant cane or stubble cane, and soil type, generalized as light or heavy textured soils, while not accounting for other crop and environmental characteristics such as crop growth conditions or crop N demand [1]. These N rate recommendations are applied in a single application from the beginning of April to the beginning of May. This N application timing provides sugarcane producers the flexibility to utilize further management techniques to accurately estimate N rate recommendations in-season, which can account for the spatio-temporal variability of sugarcane production system.

Historically, soil sampling has been a technique utilized for determining N rate recommendations. However, the reliability of soil N tests is often questionable due to the challenges associated with the dynamic nature of N in the soil, particularly in the humid alluvial soils of Louisiana [4]. Therefore, crop yield monitoring has become an important aspect of many N management schemes. A common method of incorporating crop yield into N rate recommendations is through the use of yield goals, specifically in cereal crop production [5]. A yield goal is defined as yield per unit area we might expect to achieve given adequate growing conditions and it is determined by taking a recent five year average plus 30% to account for potentially above average growing conditions. Johnson [6] and Schmitt [7] reported the importance of yield goal for N recommendations in winter wheat (*Triticum aestivum*) and corn (*Zea mays*), respectively. They indicated that 33 kg·N·ha^−1^ for every 1 Mg of wheat and 20 kg·N·ha^−1^ for every 1 Mg of corn would be required. However, setting yield goals at unrealistic levels can lead to under-or over-estimation of N rate recommendations. This is envisaged especially when N recommendations based on yield goals across large scale spatial variability do not take into account temporal variability, due to environmental growing conditions, nor within field spatial variability.

Due to limitations associated with utilizing yield goals, research in other crops such as wheat and corn has focused on in-season crop monitoring as an approach to N management. However, limited research is available for sugarcane production, particularly Louisiana sugarcane production. Additionally, research that is available has produced negative or inconclusive results [8,9]. Wiedenfeld [9] reported that chlorophyll meters were not a viable tool for predicting N recommendations for sugarcane grown in the Lower Rio Grande Valley. This lack of viability is partially due to the chlorophyll meter relying solely on plant tissue N concentrations and N accumulation in sugarcane occurred later in the season compared to when measurements were taken.

Many plant indices based on canopy spectral reflectance have shown the ability to accurately estimate crop physiological properties, including plant biomass and crop yield [10–12]. The NDVI value, which is a vegetative index that compares reflectance at the red and near infrared region, has also shown the ability to determine yield potential (YP) [13–15]. Yield potential differs from yield goal because it is a function of the environmental conditions of the current growing season and is defined as achievable yield with no additional N fertilizer [11]. Teal [14] reported that there was a strong relationship between NDVI and grain yield in corn using an exponential model. Raun [13] and Lukina [16] showed this relationship was improved when NDVI readings were adjusted using growing degree days (GDD), where NDVI was divided by GDD accumulated from planting to sensing, to create an in-season estimate of yield (INSEY). Raun [13] reported that six of nine sites over two years showed a strong relationship between INSEY and grain yield at harvest (coefficient of determination (*r^2^* = 0.83, *P* < 0.01). However, Teal [14] found there was no significant increase or decrease in the strength of this relationship when NDVI readings were adjusted by either GDD or days from planting to sensing (DFP) when GDD was positive.

Several studies have suggested that growth stage, or time of sensing, were important in the ability to predict yield [13,14,16]. Raun [13] and Lukina [16] reported that the strongest relationship between NDVI and winter wheat grain yield was between Feekes 4 to 6, while Teal [14] found that the optimum growth stage for predicting corn yield was at the eight leaf vegetative phase, or between 800–1,000 GDD. They found a weak relationship during early growth stages, which was attributed to the yield potential not fully developed. Additionally, they explained the disappearance of this weaker relationship later in the season was due to canopy closure, which resulted in the inability to detect variability associated with differing N-rates. Several reports have shown that an estimate of yield alone is poorly correlated with optimum N rate [17]. However, Raun [11] showed the potential of utilizing a predicted YP as a component of N management scheme. This technology has shown the ability to improve N management decisions in many cropping systems across USA, Canada, Mexico, and other countries [18–20]. These reports suggest the potential of using yield prediction as an integral part of an N management decision tool to improve recommendations in crop production. Previous reports have documented the ability of NDVI to estimate sugarcane yield potential, however, most of these reports have been focused on satellite based platforms or passive sensors with few demonstrating the ability of a active ground-based remote sensor to estimate sugarcane yield [21–25]. Therefore, the objectives of this study were to: (1) determine the ability of an in-season estimation of NDVI to predict sugarcane yield potential; and (2) determine optimum timing for predicting sugarcane in-season yield potential.

## 2. Experimental Section

Research was conducted in St. Gabriel (30°15′13″N 91°06′05″W) and Jeanerette (29°54′59″N 91°40′21″W), Louisiana, on several N-rate field trials. Soils utilized for each experiment are as follows: Commerce silt loam (Fine-silty, mixed, superactive, non-acid, thermic Fluvaquentic Endoaquept) for Experiments 1, 2, 3, 4, and 9; Canciene silty clay loam (Fine-silty, mixed, superactive, nonacid, hyperthermic Fluvaquentic Epiaquept) for Experiments 5, 6, 7, and 8; and Baldwin silty clay loam (Fine, smectitic, hyperthermic, Chromic Vertic Epiaqualf) for Experiments 10 and 11. Average monthly temperature and precipitation for each location and year are provided in Tables 1 and 2, respectively.

Detailed descriptions for all experiments are provided in Table 3, as well as varieties used presented in Table 3. Experiments were planted on beds in a three-row plot, measuring approximately 2 m wide. The row length of most experiments was 15 m long with the exception of Experiment 7 (13.3 m long), 6 (11.6 m long), and 9 (8 m long). Excluding Experiment 6, all experiments were planted by hand using whole stalks where open furrows where filled with stalks that were placed with an overlap of 8 cm or two matured internodes of the adjacent stalk. Experiment 6 was billet planted, using 50 cm segments of sugarcane (billets), planted at the rate of 6 billets wide within an open furrow. These billets were then planted in 50 cm sections down the planning furrow. Following planting, all rows were covered with approximately 6 cm of soil and packed firmly using a custom roller packer. Nitrogen fertilizer was knifed in the shoulder of the bed as urea-ammonium nitrate (UAN; 32-0-0) to all trials at the rate of 0, 45, 90, and 135 kg·N·ha^−1^, with the exception of Trial 2, 3, 5 (2008), and 8 which received the following N-rate: Experiment 2, received 0 and 135 kg·N·ha^−1^; Experiment 3, received 0, 45, 90 kg·N·ha^−1^; Experiment 5; received 0, 17, 67, 135, and 201 kg·N·ha^−1^; Experiment 8, received 0, 45, 90, 135, and 180 kg·N·ha^−1^. Following application of fertilizer, knife furrows were covered. Weed management was carried out according to current Louisiana State University AgCenter herbicide recommendations which included application of metribuzin (4-amino-6-*tert*-butyl-4,5-dihydro-3-methylthio-1,2,4-triazin-5-one) in early spring prior to emergence of the sugarcane crop and atrazine [2-chloro-4-(ethylamino)-6-(isopropylamino)-s-triazine] was applied when beds were rebuilt in-season (lay-by), approximately in the middle of May. Sensor readings were taken with the GreenSeeker®, an active ground-based handheld sensor (Trimble Navigation, Ltd., Sunnyvale, CA, USA), which measures canopy reflectance at the red region (670 ± 10 nm) and the NIR region (780 ± 10 nm) and NDVI was determined based on equation (1):

(1) $$\text{NDVI}=[(\rho_{\text{NIR}}-\rho_{\text{Red}})/(\rho_{\text{NIR}}+\rho_{\text{Red}})]$$

where:

*ρ*_NIR_ = reflectance at the near infrared (NIR)
*ρ*_Red_ = reflectance at the red

The GreenSeeker® sensor takes eight readings per second. For this study, the average number of readings taken per plot was between 90 and 110. Additionally, the sensor's field of view is approximately 60 cm with optimum height above the crop canopy being 80 to 110 cm. Sensor readings were taken weekly for eight weeks beginning approximately the middle to late April with 17th April being the earliest sensing date until early June with 12th June being the latest sensing date. For Experiment 6 and 8, sensor readings were taken for five consecutive weeks starting one week after fertilization, with fertilizers being applied from the middle of April until the end of May.

Plots were harvested with a Cameco C2500 chopper harvester (Cameco Industries, Thibodaux, LA, USA) and total harvested cane tonnage was determined using a weigh wagon fitted with load cells. Ten randomly selected sub-sample stalks were collected from the middle row, leaves were removed from the stalk, and each stalk was cut approximately 10 to 12 cm below the apical meristem. After weight determination, the sub-samples were analyzed for sugarcane quality parameters using a Spectracane Near Infrared System (Bruker Coporation, Billerica, MA, USA).

Prior to analysis, data were grouped by sensing date and cumulative growing degree days (CGDD) at time of sensing. Normalized difference vegetative index values were adjusted by two different methods to create an INSEY. The first normalization (INSEY-DFY) was calculated similar to [11], based on Equation (2):

(2) In‐season estimate of yield‐day of year=NDVI/DFY

where DFY = all days from the beginning of the year where GDD > 0.

Teal [14] implemented a similar index in corn by dividing NDVI values by the number of days from planting to sensing. However, since sugarcane is a semi-perennial crop and senesces during the winter, the beginning of the calendar year was used. In the second method, the plant index was determined by comparing NDVI values to the CGDD from the beginning of the year (INSEY-GDD), based on Equation (3):

(3) In‐season estimate of yield‐cumulative growing degree days=NDVI/CGDD

where:

CGDD = the cumulative growing degree days from the beginning of the calendar year.

Growing degree days were determined by the optimum day method [33], based on Equation (4):

(4) $$\text{Cumulative growing degree days}=((\text{Temp}._{\max}-\text{Temp}._{\min})/2)-\text{base temperature}$$

where:

Temp_max_ = maximum daily temperature;
Temp_min_ = minimum daily temperature;
Base temperature = 18 °C for sugarcane production.

Statistical analysis was performed using SAS software [34]. For Experiments 1 and 3, no significant effect of foliar fertilization was found; therefore, further analysis was carried out across foliar treatments. In addition, for Experiments 4, 5, 7, 9, 10, and 11 the variety by N-rate effect was not significant and analysis was carried out across varieties. Linear and non-linear regression analyses were performed in SAS to determine the relationship between NDVI, INSEY-DFY, INSEY-CGDD, and sugarcane yield components using Procedure Reg (linear regression) and NLIN (non-linear regression). Coefficient of determination (*r^2^*) values obtained from Proc Reg and NLIN were used to evaluate the models.

## 3. Results and Discussion

### 3.1. Sugarcane Yield Summary

Cane tonnage and sugar yield varied across sites and years (Table 5). Sugarcane yields in Louisiana, as well as soil properties, have been previously found to show similar variability based on crop age and growth conditions [35]. The average yield across all 12 site years was 65 Mg·ha^−1^ for cane tonnage and ranged from 31 Mg·ha^−1^ to 100 Mg·ha^−1^; additionally sugar yield averaged 7.8 Mg·ha^−1^ and ranged from 2.2 to 12.1 Mg·ha^−1^. Yield achieved by Experiment 11 in 2011 achieved the highest cane tonnage with 100 Mg·ha^−1^ and Experiment 7 in 2010 achieved the highest sugar yield with 12.1 Mg·ha^−1^. The higher yields were potentially associated initiation of rapid biomass accumulation, at which time water consumption is highest [36]. Experiment 8 in 2011 yielded the lowest cane tonnage with 31 Mg·ha^−1^ and Experiment 5 in 2009 with 2.2 Mg·ha^−1^ for sugar yield. The lowered production for both cane tonnage and sugar yields can be attributed to the increased crop age, both being 2nd stubble sugarcane crops. Johnson [35] reported that sugarcane yield typically decreased with increasing age. In addition, the lowered cane tonnage for 2011 could be attributed to a high lodging rate due to winds associated with tropical storm Lee, which made landfall during maturation on 9th September 2011. This high lodging rate can attribute to low harvest efficiency.

### 3.2. Optimum Timing for Prediction of Sugarcane Yield Potential Using NDVI

Timing of sensing is an important factor in determining the feasibility of integrating predicted YP into N management schemes. GreenSeeker® sensor readings were obtained from early April until the first of June; further sensing dates were not investigated due to the potential of physically damaging the sugarcane crop with the equipment crossing the field. Sensing dates that do not fully coincide with the existing narrow timeframe associated with in-season fertilization of sugarcane in Louisiana (1st April through 30th April) were investigated due to limited research currently available for the effects of later fertilization timings.

Sugarcane grown in Louisiana goes through four growth stages: emergence, tillering, grand growth, and maturation, each lasting from one to three months. Therefore, identifying sensing ranges based on growth stage, as proposed by several other studies, may not be feasible [11,14,16]. Overall, using DFY in which the CGDD > 0 as a measure of time of sensing resulted in weak exponential or non-significant relationships (Table 6). These weak relationships can be attributed to rapid accumulation of days in the beginning of the season, even when the weather is cooler and growth is minimal. For example, if the average daily temperature was 19 °C there would be no difference in the number of days accumulated compared to the average daily temperature of 32 °C, the latter being within optimum temperature range for sugarcane growth.

However, when CGDD was used as a measure of time, stronger exponential relationships were achieved (Table 6). All spectral reflectance measurements showed a no significant or a weak relationship for both cane tonnage and sugar yield from 150 to 600 CGDD (Table 6). Begue [23] explained variability in NDVI readings using SPOT images from early season growth. They attributed the variability to factors such as cane residues and weed development which could be highly influential at low stand densities early in the growth season.

Additionally, this weak relationship could be potentially due to lowered N uptake and YP not being fully developed at early growth. Kwong [37] reported that fertilizer N accumulation in sugarcane was low prior to a period of rapid N uptake, approximately 140 to 150 days after previous harvest. Thus differentiation in N uptake between high N rate plots and lower N rate plots would not be evident until later in the growing season. The strongest relationship occurred between 601 to 751 CGDD (Table 6). This timeframe corresponded to the last week in May to the first week in June for all years. The relationship between spectral reflectance values and sugarcane yield after 751 CGDD substantially decreased. Several reports have shown a similar trends, where the relationship between NDVI and yield increased as the crop developed [22–25]. However, a majority of these reports found higher rela tionships later in the growing season toward maturity, which this study did not investigate. One such reported was Rao *et al.* [25] which indicated the correlation between NDVI and sugarcane yield increased throughout rapid growth until the end of the grand growth stage. For this study, the grand growth phonological stage resulted from the accumulation of approximately 751 CGDD. During this stage of growth the sugarcane crop began to rapidly accumulate biomass. This increased biomass production resulted in canopy closure, decreasing the ability of NDVI to distinguish variation and therefore yield [14].

**Figure 1.**
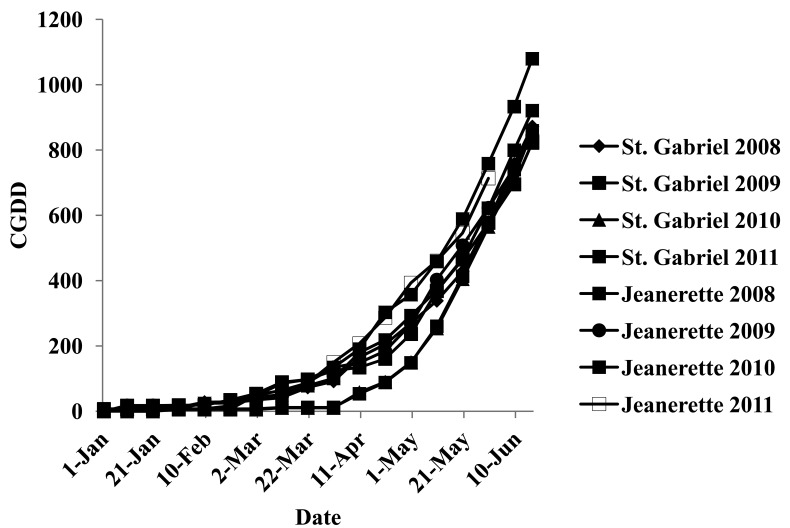
Total accumulation of growing degree days (CGDD) as a function of day of the year from the beginning of January until mid-June.

Flowers [38] reported that the application of N fertilizer when the crop is responsive, *i.e.*, during rapid accumulation, can increase crop yield and decrease loss. According to Teboh (unpublished data) the initiation of rapid N uptake was approximately 5th June for sugarcane production in Louisiana. However, N fertilization for sugarcane production in Louisiana is between 1st April and 30th April, which is approximately 100 to 275 CGDD during a normal site year (Figure 1) [1]. However, limited research has been conducted to determine the effects of delaying fertilization later into May (approximately 250 to 650 CGDD, Figure 1). Even though the effects of delaying N fertilization are unknown, these effects are influenced by environmental conditions that control natural N additions and crop response. This research indicates that delaying N fertilization is essential to integrate an in-season yield potential into sugarcane N management schemes. However, the benefits of delaying sugarcane N fertilization to coincide with optimum time for sugarcane yield prediction may not outweigh the risks involved with delayed fertilization in late May, such as yield losses from physical damage to the sugarcane by the fertilizer applicator.

### 3.3. Adjusting NDVI Readings Using DFY and CGDD

Overall, the exponential relationship measured from 601 through 750 CGDD between NDVI and sugarcane yield was low (Table 6) compared to similar models for both corn and winter wheat [14,16]. One potential factor for weaker relationship between NDVI readings and sugarcane yield was the variability of NDVI readings associated with different growing conditions between locations and years. Normalization methods have been implemented previously in an attempt to standardize the variability associated with different growing conditions [14]. Two adjustment methods were evaluated in this study, both the INSEY-DFY and INSEY-CGDD. Both adjusted methods responded similarly to NDVI as a function of time in which CGDD of 601 through 750 being the optimum time for both methods (Table 6). Table 7 reports the relationship between sugarcane yield and both adjustment methods, as well as NDVI, at the 601 to 750 CGDD stage across all varieties. The INSEY-DFY only slightly improved YP estimation compared to the unadjusted NDVI for cane tonnage (*r^2^* = 0.23 compared to 0.2 for unadjusted NDVI); however, INSEY-DFY strengthened the relationship with sugar yield compared to the unadjusted NDVI value (*r^2^* = 0.33 compared to 0.21 for unadjusted NDVI). The INSEY-CGDD adjustment improved the relationship between cane tonnage and sugar yield compared to both unadjusted and INSEY-DFY (*r^2^* = 0.48 and 0.42 for cane tonnage and sugar yield, respectively).

It has been documented that temperature significantly affects canopy development in sugarcane production [39–42]. Inman-Bamber [39] reported that moisture did not significantly impact early canopy development and only influenced the number of green leaves per stalk and final leaf area under water stressed conditions. Although INSEY-DFY was found to improve the YP estimation, especially for sugar yield, INSEY-CGDD obtained more consistent improvement across different growing conditions for both cane tonnage and sugar yield. This is because CGDD is a measure of cumulative temperature across the growing season and NDVI is a measure of crop greenness and biomass. Therefore, in highly variable conditions associated with sugarcane production in the mid-South, INSEY-CGDD adjustment would increase the stability of sugarcane yield prediction models utilized across different locations and years.

### 3.4. Separating Prediction Models Based on Canopy Structure

The canopy structure of sugarcane has been shown to be highly variable, particularly between the different varieties [43–45]. Galvao [43] further reported that spectral reflectance can be used as a tool for distinguishing different sugarcane varieties, due to difference in canopy architecture. Therefore, the accuracy of a yield prediction model based on canopy reflectance created across varieties could be lowered due to the variability associated with differing canopy structures. While having a separate YP equation for each variety would provide the most accuracy, the feasibility of creating multiple models for in-season management decision for sugarcane production would be challenging. However, a model which grouped varieties based on canopy structure would decrease the variability associated with different architectures.

**Figure 2.**
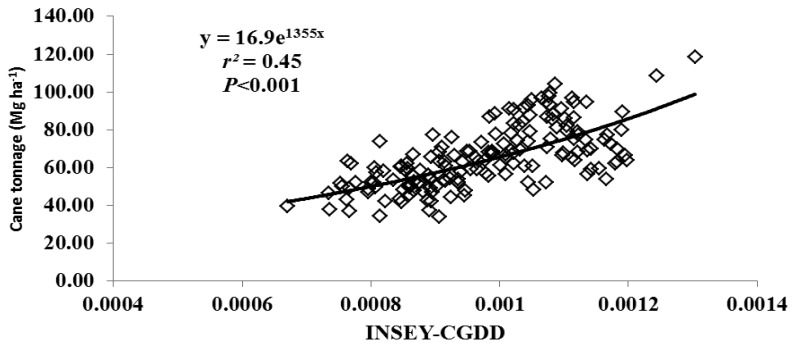
Relationship between cumulative growing degree days adjusted NDVI (INSEY-CGDD) and cane tonnage for droopy varieties (L 97-128, L 99-226, L 99-233) for all locations between 601 through 750 CGDD from 2008 to 2011 in Louisiana, USA.

**Figure 3.**
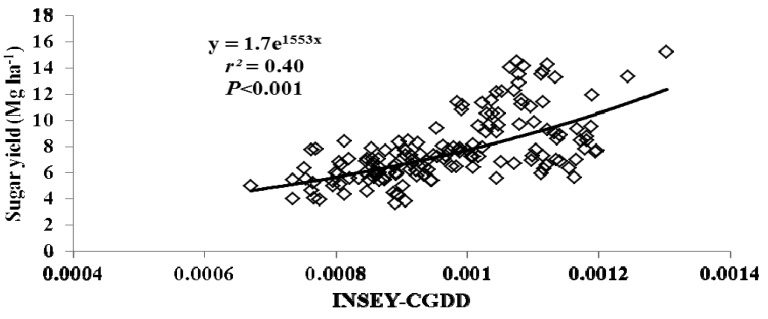
Relationship between cumulative growing degree days adjusted NDVI (INSEY-CGDD) and sugar yield for droopy varieties (L 97-128, L 99-226, L 99-233) for all location between 601 and 750 CGDD from 2008 to 2011 in Louisiana, USA.

**Figure 4.**
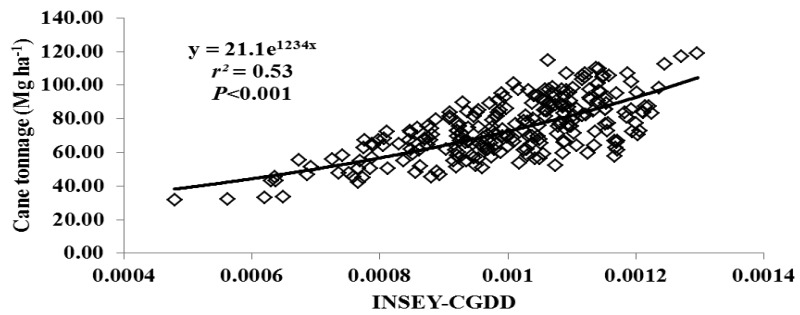
Relationship between cumulative growing degree days adjusted NDVI (INSEY-CGDD) and cane tonnage for erect varies (L 01-283, LCP 85-384, HoCP 96-540, Ho 95-988) for all locations between 601 through 750 CGDD from 2008 to 2011 in Louisiana, USA.

**Figure 5.**
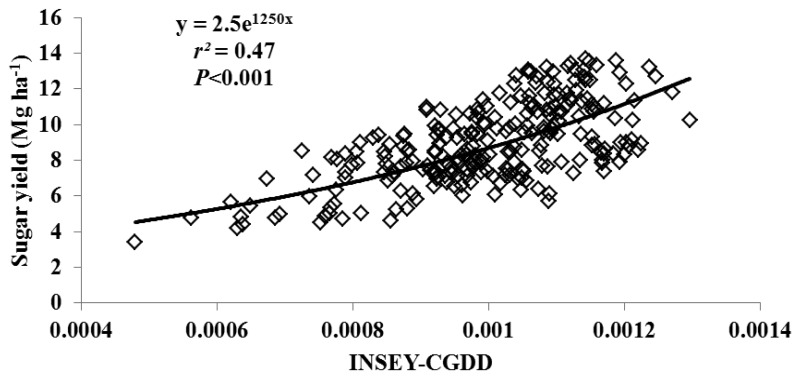
Relationship between cumulative growing degree days adjusted NDVI (INSEY-CGDD) and sugar yield for erect varies (L 01-283, LCP 85-384, HoCP 96-540, Ho 95-988) for all locations between 601 through 750 CGDD from 2008 to 2011 in Louisiana, USA.

For this study, varieties were grouped as either erectophile (erect) or planophile (droopy) based on varietal registration reports (Table 4). Figures 2 through 5 illustrate the relationship between INSEY-CGDD and sugarcane yield when varieties were separated as either droopy (Figures 2 and 3) or erect (Figures 4 and 5) when measurements were taken between 601 to 750 CGDD. The model that contained solely the erect varieties improved the YP model, with *r^2^* values of 0.53 for cane tonnage and 0.47 for sugar yield compared to 0.46 and 0.42 for cane tonnage and sugar yield, respectively of all varieties. Conversely, there was a slight reduction in the exponential relationship with models that contained only the droopy varieties, with *r^2^* values of 0.45 and 0.40 for cane tonnage and sugar yield, respectively.

This decreased exponential relationship can be attributed to droopy varieties canopy spreading wider than erect leaf canopy structure leading to canopy closure earlier in the season. Therefore, the sensor's field of view tends to be occupied more with green biomass and only limited soil background [46]. In such conditions the sensor loses its sensitivity. This situation is not the case for erect leaf-canopy structure. Separating YP models based on canopy structure increased the accuracy at which the erect varieties could be predicted; however, it decreased the YP estimation of the droopy varieties.

An N management scheme that utilizes predicted YP would allow sugarcane producers to adjust in-season N recommendations based on expected yield. Since YP is the yield expected to be achieved with no additional N fertilizer, it cannot be used independently to determine N rate recommendations. However, YP has been successfully integrated into an N management scheme which incorporates YP and a response index value to successfully estimate in-season N rate recommendations in other crops [14,47,48]. Lofton [49] reported that an in-season response index value could be successfully used to predict sugarcane yield response to applied N. To incorporate an N management decision tool that utilizes in-season estimation of YP, N fertilization would need to be delayed to coincide with the optimum timeframe for estimating YP, 601 to 750 CGDD, based on the findings of this study. The decision to delay N fertilization to coincide with in-season estimate of YP would need to be carefully evaluated on a field by field basis due to risks associated with N fertilization later in the season, including physical damage to the sugarcane by mechanically passing through the field. Additionally, due to chemical and physical factors that could influence the accuracy of YP estimations, YP should be determined separately for each management zone across the field. By using an N management scheme which takes into account YP, sugarcane producers can take advantage of years in which N demand may be higher or lower due to other yield limiting or enhancing factors.

## 4. Conclusions

This study demonstrated that NDVI readings can be used to estimate in-season sugarcane YP. The use of DFY did not provide positive results as a measure of time because of rapid accumulation of days early in the growing season when growth is minimal. The optimum timeframe for estimating sugarcane YP was determined to be from 601 through 750 CGDD. Because this timeframe is outside the current recommendations for N fertilization, sugarcane producers would need to delay in-season N fertilization by one month in order to integrate yield potential into an N management scheme. The risks and benefits of adopting this N management scheme would need to be evaluated on a producer basis.

Adjusting NDVI readings using CGDD and DFY increased the accuracy of YP estimation models but only CGDD adjustment increased the relationship between NDVI and cane tonnage. The CGDD adjustment provided a better prediction of sugarcane YP because it provided a better estimation of temperature throughout the growing season compared to DFY. Additionally, separating varieties based on canopy structure increased the *r*^2^ value of the YP model with the varieties that were classified as erect; however, it had a slightly negative effect on the relationship between canopy reflectance and sugarcane yield for the droopy varieties. This was due to increased canopy closure early in the growing season of the droopy varieties. This increased green vegetation and decreased soil background diminished the sensitivity of the sensor to detect canopy variability associated with different N treatments. Therefore, when an N management system which integrates in-season predicted yield potential is implemented, sugarcane producers need to be aware of both the CGDD throughout the growing season, because this is utilized as a time-frame for when to collect NDVI readings and an adjustment method for NDVI values, and sugarcane variety, due to differences between varieties associated with different canopy structures. An important concept to realize is the scale of this technology is much finer that those discussed in previously literature which have been based on satellite platforms. This finer scale allows for the detection of variability within or between management zones; however, limits its use on a regional scale, compared those satellite platforms.

Further research is needed to develop specific guidelines for distinguishing different canopy structures. In this study, the authors utilized variety reports to determine differences in canopy structure; however, numerical guidelines that take into account physiological characteristics of each variety, such as leaf angle or length of leaf to the first bend, would provide a more precise method of separating sugarcane varieties. Additionally, continued updates will be essential to increase the robustness of this sugarcane YP model. These updates will need to incorporate new commercially available varieties as they become available and additional diverse growing conditions. Also, due to limitations associated with NDVI, additional plant indices evaluation would be beneficial, this is especially true for those indices that are less prone to saturation at high biomass situations, such as with the droopy varieties.

## Figures and Tables

**Table 1.** Average monthly temperature (°C) observed in 2008–2011 for St. Gabriel and Jeanerette, LA.

**Month**	**St. Gabriel, LA, USA**				**Jeanerette, LA, USA**			

	**2008**	**2009**	**2010**	**2011**	**2008**	**2009**	**2010**	**2011**
January	10.4	11.9	8.2	4.8	11.2	9.0	8.9	6.3
February	14.1	13.9	7.7	8.5	15.0	11.1	8.4	8.9
March	16.0	17.7	13.3	13.8	16.8	14.2	13.7	14.9
April	19.7	19.2	20.1	18.8	20.5	17.4	20.9	19.7
May	24.1	24.2	25.6	21.0	24.4	22.8	26.0	21.7
June	27.4	27.9	27.9	26.1	27.6	25.7	28.4	25.6
July	24.5	28.5	28.4	26.5	28.2	25.9	28.6	N/A †
August	27.2	27.2	28.5	26.7	27.6	25.3	28.9	N/A
September	24.7	26.0	26.1	21.8	25.2	23.4	26.8	N/A
October	18.9	20.1	19.9	14.0	20.0	19.0	20.7	N/A
November	14.1	14.3	15.4	12.6	15.3	11.2	16.6	N/A
December	13.2	10.4	10.6	12.2	13.8	10.9	10.9	N/A

† Indicates the information is not available for this month due to malfunctioning weather sensors.

**Table 2.** Average monthly precipitation (cm) observed in 2008–2011 for St. Gabriel and Jeanerette, LA.

**Month**	**St. Gabriel, LA, USA**				**Jeanerette, LA, USA**			

	**2008**	**2009**	**2010**	**2011**	**2008**	**2009**	**2010**	**2011**
January	14.1	10.6	6.4	4.7	15.3	5.0	5.2	8.1
February	7.6	4.5	19.9	4.5	5.3	4.9	15.6	3.5
March	2.6	18.4	6.2	9.4	5.1	23.7	4.3	7.0
April	2.2	6.8	2.3	3.2	6.0	11.4	3.0	1.2
May	13.5	2.4	15.3	0.6	13.1	9.4	8.8	1.0
June	4.4	0.7	27.1	13.0	1.9	3.5	18.2	N/A †
July	5.7	7.9	10.9	13.2	3.2	9.5	38.1	N/A
August	23.7	9.0	24.9	N/A	8.8	11.3	17.8	N/A
September	9.9	11.7	2.7	N/A	31.1	25.3	7.0	N/A
October	0.9	31.4	1.7	0.8	0.6	35.1	4.3	N/A
November	3.5	2.6	16.4	N/A	5.6	3.4	16.8	N/A
December	13.8	41.2	6.7	2.3	7.1	46.9	7.2	N/A

† Indicates the information is not available for this month due to malfunctioning weather sensors.

**Table 3.** Agronomic practices for all experiments established at St. Gabriel and Jeanerette, LA, USA from 2008 through 2011.

**Experiment No.**	**Year**	**Crop**	**Description**	**Location**	**Planting Date**	**Spring Fertilization Date**	**Harvest Date**
1	2008	2^nd^ Stubble ‡	Foliar fertilization × N rate	St. Gabriel, LA,	August 2006	15 April 2008	27 October 2008
2	2008	2^nd^ Stubble	N Response Study	St. Gabriel, LA	August 2006	15 April 2008	27 October 2008
3	2008	1^st^ Stubble	Foliar fertilization × N rate	St. Gabriel, LA	August 2007	15 April 2008	4 November 2008
	2009	2^nd^ Stubble	Foliar fertilization × N rate	St. Gabriel, LA	August 2007	15 April 2009	4 November 2009
4	2008	1^st^ Stubble	Variety × N rate	St. Gabriel, LA	August 2006	17 April 2008	5 November 2008
	2009	2^nd^ Stubble	Variety × N rate	St. Gabriel, LA	August 2006	29 April 2009	4 November 2009
5	2008	Plant Cane	Variety × N rate	St. Gabriel, LA	September 2007	14 April 2008	17 November 2008
	2009	1^st^ Stubble	Variety × N rate	St. Gabriel, LA	September 2007	6 April 2009	18 November 2009
6 †	2010	Plant Cane	N rate × N timing	St. Gabriel, LA	September 2009	15 April 2010	8 December 2010
	2010	Plant Cane	N rate × N timing	St. Gabriel, LA	September 2009	29 April 2010	8 December 2010
	2010	Plant Cane	N rate × N timing	St. Gabriel, LA	September 2009	13 May 2010	8 December 2010
	2010	Plant Cane	N rate × N timing	St. Gabriel, LA	September 2009	27 May 2010	8 December 2010
	2011	1^st^ Stubble	N rate × N timing	St. Gabriel, LA	September 2009	13 April 2011	8 December 2011
	2011	1^st^ Stubble	N rate × N timing	St. Gabriel, LA	September 2009	23 April 2011	8 December 2011
	2011	1^st^ Stubble	N rate × N timing	St. Gabriel, LA	September 2009	11 May 2011	8 December 2011
	2011	1^st^ Stubble	N rate × N timing	St. Gabriel, LA	September 2009	25 May 2011	8 December 2011
7	2010	Plant Cane	Variety × N rate	St. Gabriel, LA	September 2009	22 April 2010	22 November 2010
	2011	1^st^ Stubble	Variety × N rate	St. Gabriel, LA	September 2009	13 April 2011	3 November 2011
8 †	2011	2^nd^ Stubble	N rate × N timing	St. Gabriel, LA	September 2007	13 April 2011	13 October 2011
	2011	2^nd^ Stubble	N rate × N timing	St. Gabriel, LA	September 2007	23 April 2011	13 October 2011
	2011	2^nd^ Stubble	N rate × N timing	St. Gabriel, LA	September 2007	11 May 2011	13 October 2011
	2011	2^nd^ Stubble	N rate × N timing	St. Gabriel, LA	September 2007	25 May 2011	13 October 2011
9	2011	Plant Cane	Variety × N rate	St. Gabriel, LA	September 2010	13 April 2011	1 December 2011
10	2008	2^nd^ Stubble	Variety × N rate	Jeanerette, LA	August. 2006	25 April 2008	13 November 2008
11	2010	Plant Cane	Variety × N rate	Jeanerette, LA	November 2009	23 April 2010	17 November 2010
	2011	1^st^ Stubble	Variety × N rate	Jeanerette, LA	November 2009	11 April 2011	18 October 2011

† Four values are for the different spring N fertilization times, which yield was calculated separately for each timing;

‡ Stubble crop indicates the crop grown after the first year's harvest.

**Table 4.** Varieties used in all experiments, canopy designation, and source of canopy designation from 2008 to 2011 in St. Gabriel and Jeanerette, LA, USA.

**Experiment No.**	**Variety**	**Canopy Structure**	**Reference**
1	Ho 95-988	Erect	Tew [26]
2	L 97-128	Droopy	Gravois [27]
3	L 97-128	Droopy	Gravois [27]
4	L 99-226	Droopy	Bischoff [28]
	L 99-233	Droopy	Gravois [29]
5	L 99-226	Droopy	Bischoff [28]
	LCP 85-384	Erect	Milligan [30]
	HoCP 96-540	Erect	Tew [31]
6	L 01-283	Erect	Gravois [32]
7	L 99-226	Droopy	Bischoff [28]
	L 01-283	Erect	Gravois [32]
	HoCP 96-540	Erect	Tew [31]
8	L 97-128	Droopy	Gravois [27]
9	L 99-226	Droopy	Bischoff [28]
	L 01-283	Erect	Gravois [32]
	HoCP 96-540	Erect	Tew [31]
10	Ho 95-988	Erect	Tew [26]
11	L 99-226	Droopy	Bischoff [28]
	L 01-283	Erect	Gravois [32]
	HoCP 96-540	Erect	Tew [31]

**Table 5.** Average sugarcane yield at different nitrogen fertilization rates achieved from 2008–2011 from St. Gabriel and Jeanerette, LA, USA.

		**Cane Tonnage**				**Sugar Yield**			

**Experiment No.**	**Year**	**0N** †	**45**	**90**	**135**	**0N**	**45**	**90**	**135**
		Mg·ha^−1^							
1	2008	68	71	73	71	8.53	8.76	9.06	8.68
2 ‡	2008	39	-	-	68	5.21	-	-	7.54
3 ‡	2008	71	74	77	-	8.72	8.94	9.20	-
	2009	54	53	55	-	6.15	6.07	6.36	-
4	2008	56	62	63	61	6.87	7.34	7.35	7.17
	2009	51	69	76	75	5.13	7.13	7.65	7.49
5 §	2008	83	75	85	83	10.53	9.46	10.49	10.25
	2009	49	54	48	53	2.25	2.52	2.82	3.16
6 ¶	2010	97	88	89	91	12.45	11.43	11.40	11.66
	2011	58	66	70	68	10.09	8.69	9.04	8.82
7	2010	83	90	85	91	10.20	11.95	12.87	13.27
	2011	46	62	79	77	5.64	7.82	10.00	9.68
8	2011	39	43	41	41	4.41	4.62	4.40	4.34
9	2011	81	83	86	90	10.20	10.50	10.80	11.40
10 #	2008	31	51	53	44	4.19	6.79	7.35	6.00
11	2010	66	68	70	65	8.26	8.38	8.98	7.86
	2011	83	92	85	100	10.83	11.22	10.61	11.37

† Indicate applied N rates in kg·N·ha^−1^;

‡ Data points were not available due to particular plots did not receive designated N rates;

§ N rates used were 0, 17, 67, 135, and 201 kg·N·ha^−1^. Yield values for the 45 and 90 kg·N·ha^−1^ columns were plots which received 17 and 67 kg·N·ha^−1^, respectively. Additionally 201 kg·N·ha^−1^ yielded 83 MT ha^−1^ and 10463 kg·ha^−1^ for cane tonnage and sugar yield, respectively;

¶ Indicate a significant response (*P* < 0.05); however, the highest significant yield was the check plot;

# Additionally 180 kg·N·ha^−1^ yielded 64 Mg·ha^−1^ and 8.8 Mg·ha^−1^ for cane tonnage and sugar yield, respectively.

**Table 6.** Exponential relationship between spectral reflectance measurements and sugarcane crop yield component as a function of time in St. Gabriel and Jeanerette, Louisiana from 2008 through 2011, using cumulative growing degree days (CGDD) and day from the beginning of the year (DFY) when growing degree days > 0.

	
	Coefficient of Determination (*r^2^*)					
	
	Cane Tonnage			Sugar Yield		
Growth Stage	NDVI	INSEY-DFY ^†^	INSEY-CGDD ^‡^	NDVI	INSEY- DFY	INSEY-CGDD
**CGDD**						
150–300	NS §	NS	NS	NS	NS	NS
301–450	NS	NS	NS	0.30	NS	0.25
451–600	0.24	NS	0.24	0.22	NS	NS
601–750	0.20	0.23	0.46	0.21	0.33	0.42
>751	NS	0.19	0.31	0.15	0.19	0.22
**DFY**						
116–123	NS	NS	NS	NS	NS	0.10
124–131	NS	NS	NS	NS	NS	NS
132–139	0.07	NS	NS	0.11	NS	NS
140–147	NS	NS	0.21	0.11	0.15	0.34
148–155	NS	0.05	0.21	NS	NS	0.15
156–163	NS	0.25	0.31	NS	0.28	0.34

† NDVI measurement adjusted for days from beginning of year (DFY) where the growing degree days are >0;

‡ NDVI measurement adjusted for cumulative growing degree days (CGDD);

§ Indicates the relationship was not significant at a 0.05 level.

**Table 7.** Coefficient of determination (*r^2^*), equation, and *P*-value for relationship between NDVI, INSEY-DFY, and INSEY-CGDD with sugarcane yield component fit with an exponential relationship at 650 through 750 CGDD.

**Plant index**	**Cane tonnage**			**Sugar yield**		

	***r^2^***	**Equation**	***p*-value** †	***r^2^***	**Equation**	***p*-value**
NDVI	0.20	y = 25.2e^1.5x^	0.014	0.21	y = 2.9e^1.5x^	0.025
INSEY-DFY	0.23	y = 39.5e^59.2x^	<0.001	0.33	y = 3.6e^87.3x^	<0.001
INSEY-CGDD	0.46	y = 18.9e^1303x^	<0.001	0.42	y = 2.1e^1390x^	<0.001

† *p*-values are for overall models.
